# ‘Trials and Tribulations’: The Ambivalent Influence of Temporary Accommodation on Mental Health Recovery in Chronically Homeless Adults

**DOI:** 10.1177/10497323221147127

**Published:** 2023-01-16

**Authors:** Dimitar Karadzhov

**Affiliations:** 1 Centre for Health Policy, School of Social Work and Social Policy, 3527 University of Strathclyde, Glasgow, UK

**Keywords:** recovery, mental health, homelessness, temporary accommodation, qualitative

## Abstract

Relatively few studies have explicitly examined whether and how shelter-type, temporary or emergency accommodation shapes homeless clients’ personal (mental health) recovery. A transatlantic phenomenological qualitative study was conducted to examine the influence of those services on personal recovery. Eighteen chronically homeless adults with a history of serious mental illness were recruited from several temporary accommodation services in New York City (NYC), U.S., and Glasgow, Scotland. Participants completed repeat in-depth interviews and a novel one-week multimedia mobile phone diary. The interpretative phenomenological analysis (IPA) produced three overarching group experiential themes: ‘*everything was just starting to fall into place’; ‘caught in a trap’*; and ‘*trials and tribulations*’. Collectively, the findings underscore the duality of influence of temporary accommodation on recovery. Those ambiguous spaces confronted participants with existential uncertainty, volatility and chronic boredom, but also proffered opportunities for envisioning and enacting recovery. Embarking on recovery while residing in temporary accommodation is possible, even for those enduring chronic life adversity. However, it is contingent upon enabling socio-material, affective and relational resources. Implications are discussed for theorising recovery as a contextually embedded, relational phenomenon, and for providing recovery-oriented support across the housing continuum.

## Introduction

Originating from the psychiatric survivor movement, personal recovery denotes the individual’s unique journey of regaining a meaningful, satisfying and self-directed life despite the chronic nature of their mental health difficulties (Anthony, 1993; Leamy et al., 2011). Often described as a non-linear journey of personal exploration, risk-taking and self-discovery, personal recovery reflects an individual’s own biography, goals and values, inner strengths and vulnerabilities (Slade et al., 2008). As such, it may entail the acquisition of knowledge, skills, relationships, autonomy and a sense of purpose to enable control over one’s mental illness symptoms, social integration, vocational realisation and other personally valued outcomes (Leamy et al., 2011; SAMHSA, 2020; Tew et al., 2012).

In recent years, the core corpus of personal recovery scholarship has been criticised for failing to account for the multitude of socio-structural, relational and socio-cultural contexts shaping individuals’ recovery journeys (Padgett et al., 2016; Price-Robertson et al., 2017; Stuart et al., 2017). This ‘silencing’ of context, critics argue, perpetuates an individualistic notion of recovery, whereby the burden of getting well and leading a self-directed life is placed solely on the individual (Stuart et al., 2017). Subsequent empirico-theoretical work has emphasised the contextual, dynamic and relational nature of recovery (Doroud et al., 2018; Duff, 2016; Price-Robertson et al., 2017). Particularly, research into the ‘*emplacement*’ of recovery offers promise for reconceptualising recovery processes such as hope, meaning-making and social connectedness as grounded in material, relational and socio-structural realities (Duff, 2016; Piat et al., 2017). Fundamentally, Duff (2010) proposes that *place* ought to be more aptly conceptualised not merely as a collection of material and physical features but rather as ‘[…] *a social and relational production involving diverse material, social and affective elements.*’ (p. 338). Such theorising provides impetus for exploring recovery in previously neglected settings.

Examining personal recovery in individuals with a history of homelessness offers the opportunity to unravel how individuals’ recovery capacities are shaped by socio-economic disadvantage and other significant life adversity (Padgett et al., 2016). The following sections trace the landscapes of precarity homeless persons navigate in the U.S. and Scotland, before discussing the empirical literature on recovery and emergency and other temporary homeless accommodation.

### Landscapes of Precarity: Homelessness and Temporary Accommodation in NYC and Scotland

The U.S. Substance Abuse and Mental Health Services Administration (SAMHSA, 2020) highlights *home* – ‘*having a stable and safe place to live*’ – as one of the pillars of recovery. Homelessness, therefore, fundamentally impedes personal recovery (Bonugli et al., 2013; Kerman & Sylvestre, 2020; Padgett et al., 2016). The challenges to initiating and sustaining recovery are most severe for chronically homeless persons who have a history of serious mental illness (SMI) and/or substance use (Farrell, 2012; Olivet et al., 2010; Padgett et al., 2016). Chronic homelessness has been defined as having *‘[...] a disability* [and having been] *continuously homeless for one year or more or* [having experienced] *at least four episodes of homelessness in the last three years where the combined length of time homeless in those occasions is at least 12 months*’ (U.S. Department of Housing and Urban Development, 2017, p. 2). A common disability among this group, SMI refers to ‘[...] *a diagnosable mental, behavior, or emotional disorder that causes serious functional impairment that substantially interferes with or limits one or more major life activities*’ in those aged over 18 (SAMHSA, 2022). Constituting over 25% of the homeless population in the U.S., chronically homeless individuals face disproportionately higher rates of mortality, disability and unemployment, and poorer access to health and social services (Coalition for the Homeless, 2020; Farrell, 2012; Olivet et al., 2010; U.S. Department of Housing and Urban Development, 2021).

Scotland has also had a persistently high co-occurrence of repeat homelessness and mental health problems (Watts et al., 2021). During the 2020/21 period, approximately 30% of those assessed to be homeless or likely to become homeless in Scotland reported having a mental health problem (Scottish Public Health Observatory, 2021a, 2021b).

In both Scotland and NYC, a significant proportion of homeless individuals face extended stays in temporary accommodation. This includes emergency or night shelters (facilities whose key purpose is to provide temporary shelter); ‘safe havens’ (in NYC; temporary shelter services with less stringent regulations designed for hard-to-reach homeless persons); hostels and social sector accommodation (in Scotland); and transitional housing programmes (facilities offering shelter and support services for up to 24 months; U.S. Department of Housing and Urban Development, 2017; Watts et al., 2018). In NYC, under the prevailing ‘staircase model’, individuals are expected to progress through several short-term housing provisions such as shelters and transitional housing before ‘graduating’ to independent, permanent tenancies after being assessed as ‘housing-ready’ (Williams, 2017). This is usually dependent on clients’ demonstration of self-sufficiency, treatment adherence and behavioural and emotional stability. This model is characterised by conditionality, strict surveillance and long waiting lists (Williams, 2017). In the city, single adults spend an average of 431 days in large congregate shelters (NYC Government, 2020).

In Scotland, the number of households residing in temporary accommodation, mainly in the social sector, increased in 2020/21 to just over 13 000 (Scottish Government, 2021). The average length of stay in temporary accommodation in the country increased to almost 200 days in the same period (Scottish Government, 2021). In both settings, this problem has been perpetuated by temporary accommodation facilities such as night shelters, hostels and congregate emergency housing deemed ‘*not fit for purpose*’ (Watts et al., 2018: p. 14), as well as by shortages in affordable and supportive housing (Clarke et al., 2020; Padgett, 2020).

### Mental Well-Being, Recovery and Temporary Accommodation

Compared to the research documenting clients’ recovery trajectories *post-rehousing* (for example, in programmes such as Housing First; Padgett et al., 2013; Piat et al., 2017), research exploring the situated experiences of *current* homelessness and life in temporary accommodation has been markedly scarcer (Iaquinta, 2016). While the bulk of empirical work has predominantly evidenced negative shelter experiences such as victimisation, humiliation and the lack of autonomy (Kerman et al., 2019; Meanwell, 2012), other research has offered more nuanced insights, with some studies emphasising the positive effects of such services (Padgett et al., 2022). Variations in the policy context, service type, participants’ complexity of need, recruitment procedures and focus of the inquiry may have accounted for the lack of consensus about how mental well-being and recovery are shaped by temporary accommodation provisions.

To demonstrate, in an interview-based qualitative study with ‘safe haven’ clients with a history of SMI and problem substance use, Lincoln et al. (2009) explored their experiences at this service and post-rehousing in Boston, the U.S. The findings reveal clients’ expanding autonomy, independence and a sense of security and ownership as a result of the flexible, dignifying and homelike service provision. The negative aspects, however, mostly concerned the practical aspects of shelter life, with limited consideration being given to the psychological and socio-emotional challenges often faced by people with histories of homelessness, interpersonal trauma and loneliness (Kerman & Sylvestre, 2020; O'Shaughnessy & Greenwood, 2021). Also, while Lincoln et al.’s study provides empirical support for the ‘safe haven’ model, it does not explicitly address the relationship between this service and clients’ mental health recovery.

In contrast, Kerman and Sylvestre (2020) directly map experiences with a wide range of health and social services onto recovery components in their study with currently and formerly homeless adults in Canada. The currently homeless participants reported both positive (e.g. fostering social connections) and negative experiences (e.g. denigration and dehumanisation by providers; and the lack of autonomy as a result of rigid rules and ineffective communication) with accommodation-type services. Relatedly, Bonugli et al.'s (2013) study on women’s experiences of homelessness, SMI and shelters in the southwestern U.S. concluded that, despite the burden of long-term homelessness and other significant life adversity, ‘*positive growth can occur*’ (p. 834). Altogether, this body of work underscores the need for research in other jurisdictions to unravel the recovery-impeding and recovery-enabling aspects of homeless services.

### Study Aims

Relatively few empirical qualitative studies have explicitly examined whether and how shelter-type, temporary or emergency accommodation shapes homeless clients’ mental health recovery (Kerman & Sylvestre, 2020). To fill this gap, the present study aimed to explore the impact of temporary accommodation and its associated provisions on mental health recovery in chronically homeless adults with SMI in NYC, U.S., and Glasgow, Scotland.

## Methods

### Study Design, Sampling and Recruitment

A transatlantic phenomenological qualitative investigation was conducted in five emergency/temporary accommodation settings in Glasgow (Scotland) and New York City (U.S.) in 2018. An intensity sampling strategy was used to recruit clients with complex needs residing in accommodation services that specifically catered to adults deemed ‘vulnerable’ and ‘difficult-to-engage' in traditional shelter-type services (Patton, 1990; Robinson, 2014). A sample size of 18 was considered appropriate in light of the idiographic focus of this study, the heterogeneous sample, and the large amount of data anticipated from the repeat interviews and multimedia diary – enhancing data adequacy (Smith et al., 2022; Vasileiou et al., 2018). Although interpretative phenomenological analysis (IPA) is typically conducted with rather homogeneous samples (Smith et al., 2022), existing IPA studies with larger, more diverse samples have demonstrated usefulness for exploring multifaceted and underresearched phenomena (Bond et al., 2015; Vasileiou et al., 2018). Recruiting participants from multiple settings also enables the corroboration of themes across contexts – enhancing analytical rigour (Tracy, 2010).

Eligible service clients needed to be adults who spoke English fluently, had the capacity to provide informed consent and had a history of chronic homelessness and SMI. Several measures were in place to ensure non-coerciveness. First, providers ensured clients approached about this study were not experiencing acute mental health or other life crises. Second, prior to each interview, the researcher had an informal conversation with on-site staff to ensure the participant was capable of providing fully informed consent on that day; for example, to ascertain they were not visibly under the influence of alcohol. Participation was subject to written consent. Participants were offered a £15/$20 shopping voucher per interview. Ethics approval was granted by the University of Strathclyde University Ethics Committee on July 28, 2017.

### Settings

Five sites were included: two emergency accommodation services in Glasgow, and two temporary supportive housing facilities (‘safe havens’) and a drop-in centre in NYC. The NYC sites were two temporary supportive housing facilities (50–75 beds), called ‘safe havens’ – an alternative to mainstream shelters in the city, characterised by lessened, or lack of, curfews, less strict sobriety policies, and unlimited stays – and one 24/7 drop-in centre for street homeless adults (NYC Mayor’s Office of Operations, 2017). Although the drop-in centre did not provide accommodation, it offered a range of other essential facilities, including spaces for respite and socialisation. The Glasgow sites included two emergency accommodation providers – an emergency access service for women (10–30 beds) and an emergency assessment centre for men (40–70 beds). All providers offered multidisciplinary in-house support and referrals, and assisted clients with transitioning into independent housing.

### Data Collection

The multi-modal data collection included in-depth life story interviews, a novel mobile phone diary and an elicitation interview (Clark & Morriss, 2017; Gubrium & Holstein, 2001; Hall & Powell, 2011). The in-depth interview allows researchers to examine the complexities and subtleties of participants’ idiosyncratic meaning-making and thus understand what really matters to them (Pain, 2012; Smith et al., 2022). Mobile phone diaries are non-intrusive, user-friendly, efficient and adaptive approaches for capturing dynamic information about individuals’ daily lives and experiences (Karadzhov, 2021). The use of participant-generated multi-modal data and an elicitation interview aimed to stimulate participants’ recall, self-expression and reflection, and ownership of the data (Clark & Morriss, 2017; Padgett et al., 2013; Pain, 2012).

#### Procedure

During the two life story interviews, which typically occurred over two consecutive days, participants were queried about their most significant life events and experiences; contact with services; present-day life routines; and enablers and hinderers of mental well-being and recovery (Gubrium & Holstein, 2001; Hall & Powell, 2011). The first interview gathered participants’ demographic and housing history information, before exploring their early life experiences, precipitants, onset and impact of homelessness, moments of hardship and moments of joy, contact with support services, and other memorable experiences (e.g. *‘Tell me about the most significant ‘chapters’ of your life that led to your life ‘now’*.’; ‘*What significant events took place within those episodes? How did they affect you?*’; ‘*How has your life changed since you began experiencing homelessness? And what about your well-being/mental health?*’). The second interview completed the main ‘chapters’ of participants’ past, and proceeded to explore their present-day lives, including living conditions, occupations, social relationships, sources of hardship and resilience, and mental well-being and recovery (e.g. ‘*Tell me about your life now. Where do you live? What does your typical day look like?’;* ‘*What is the hardest thing about your life right now?*’; ‘*What satisfactions can you find in your life?*’).

At the end of the second interview, participants were invited to complete a seven-day smartphone app-based diary and trained on using the free EthOS diary app (https://ethosapp.com/). Using their own smartphone device or one provided by the researcher, they were asked to respond to seven daily questions and prompts via the app interface: ‘*Tell me about your day. What did you do? Where did you go?*’; ‘*Was today a good, bad or an ‘OK’ day for you*?; ‘*Show me where you spend most of your time these days.*’; ‘*Take a photo of something that best captures your life now*.’; ‘*Show me or tell me about something that is important for you at present*.’; ‘*What is something that helps you get by or improves your situation?*’; and ‘*What is something that makes your situation worse?*’. They could complete the questions in any order and at any time during the logging period. They could respond using text, a video-recording, an audio-recording or a photograph (See Karadzhov, 2021).

After the seven-day logging period, participants were invited for a final, elicitation interview to discuss the entries made (Padgett et al., 2013). The researcher presented participants with transcripts of their text, audio and video entries, and print-outs of the photographs, and asked them to provide context and discuss their significance (e.g. ‘*What is happening in this picture?*’; ‘*Do you remember what you were doing when you took this picture?*’; ‘*What does this picture tell us about your life?*’; ‘*How does this photo relate to your recovery?*’; Horwitz, 2012; Andonian & MacRae, 2011). Participants were asked to provide written consent granting the researcher permission to re-use the photographs and other diary entries. All interviews were audio-recorded and transcribed verbatim.

### Data Analysis

The interview and mobile phone diary data were analysed using IPA, following Smith et al. (2022). All interview transcripts and mobile diary images, text entries and transcripts of audio and video entries were imported into NVivo 11 (QSR International, 2015). The use of qualitative data analysis software facilitated the coding, storage and comparison of the large amounts of data. Particularly, it allowed for all interview and mobile phone diary data to be imported into each participant’s case file and analysed together.

‘Immersion’ into the data was facilitated by reading and re-reading the material empathetically, making only tentative, exploratory notes (Smith et al., 2022). Then, line-by-line coding of all data was carried out for both manifest (descriptive) and more latent (conceptual) meanings. Following Rose (2016: p. 325) and Piat et al. (2017), the participant-generated images were not treated as independent data but were coded and interpreted after the participants had discussed their meaning and significance during the elicitation interviews. Then, the researcher assigned one or more codes to each image on NVivo 11, while remaining attentive to the function the images had in each participant’s account (Rose, 2016). For example, some photographs appeared to signify absences (for instance, absence of social relationships, absences of occupational opportunities or absences of hope); others seemed to act as inventories of what participants were grateful for and cherished. The researcher (re-)interpreted the meaning of the images in light of participants’ own interpretations and in the context of the entire dataset.

For each participant, individual codes from the textual and visual data were grouped together and formed *experiential statements* capturing essential aspects of their lived experience (Smith et al., 2022). These were then grouped into higher-order clusters known as *personal experiential themes* for each participant (Smith et al., 2022). The aforementioned steps were repeated for each participant, which was followed by the identification of patterns across the entire dataset, until a final set of *group experiential themes* was developed (Smith et al., 2022). Those themes capture the most salient shared aspects across participants (Smith et al., 2022). Theoretical concepts (for example, from nursing, psychology and philosophy) were carefully ‘allowed’ into the last stage of the analysis and the write-up (Smith et al., 2022). As assured by Smith et al. (2022), an engagement with theory does not violate the idiographic tenet of IPA so long as it stays grounded in the primary data. Consistent with Larkin and Thompson (2012, p. 110–111), theory was not used to ‘*explain away’ the data*’, but rather to achieve ‘*a richer, more insightful*’ interpretation.

## Results

### Participant Characteristics and Study Engagement

Eighteen participants (ten in NYC and eight in Glasgow; 14 male and four female) were recruited. The sample consisted of nine White/Caucasian, four African-American; three Hispanic and two Asian individuals, with a mean age of 48 (range = 29–66; (one undisclosed). Fifteen participants resided in temporary accommodation (in single-occupancy rooms, dormitory-type communal rooms or apartments), while three (all in NYC) attended the 24-hour drop-in centre. Participants’ mean length of time spent homeless in their lifetime was 11 years (median = 6 years; range = 2–30 years). Twelve (67%) also reported a history of problem substance use. Their average length of stay at the current provider was approximately 10 months (Note: one undisclosed).

Altogether, 45 interviews were completed, and more than 200 diary entries were made. Nine (50%) participants completed all stages of the study. Seven of those completed the expected number of interviews – three. By exception, two participants completed four interviews because one preferred shorter interviews, and the other offered a particularly detailed life story account. Seven (39%) participants took part in only two interviews; and two (11%) participants dropped out after one interview (See ‘Table 1’). Reasons for attrition included relocation to another facility, medical emergencies, personal crises, lack of time, loss of contact and physical disabilities.

**Table 1.** Participant Profiles, Study Engagement and Theme Prevalence.

Demographic and Study Engagement Profiles						‘Everything was Just Starting to Fall into Place’: Recovery-Enabling Role of Temporary Accommodation			‘Caught in a Trap’: Recovery-Impeding Role of Temporary Accommodation			‘Trials and Tribulations’: Temporary Accommodation as an Ambivalent Site for Recovery
Participant Pseudonym (*N* = 18)	Setting	Gender	Ethnicity	Number of Interviews (*N* = 45)	Mobile Diary (*N* = 9)	‘Peace of Mind’: Restoring ‘Homelikeness’	A place for Reflection and Insight	Occupational and Social Engagement	‘Open Prison’	A Place of Illness	‘Existential Vacuum’	‘Trials and Tribulations’
Liam	U.S.	Male	Hispanic	2	No	✓		✓				
Scott	U.S.	Male	Caucasian	3	Yes	✓	✓	✓	✓		✓	✓
Matthew	U.S.	Male	Asian	4	Yes					✓	✓	✓
George	U.S.	Male	Hispanic	2	No		✓	✓				✓
Joshua	U.S.	Male	African-American	1	No		✓					
Benjamin	U.S.	Male	African-American	4	Yes	✓	✓	✓				✓
Oliver	U.S.	Male	African-American	3	Yes		✓	✓		✓		✓
Susan	U.S.	Female	Asian	2	No	✓	✓	✓		✓		
Kelly	U.S.	Female	African-American	3	Yes		✓			✓		✓
Edward	U.S.	Male	Hispanic	3	Yes	✓		✓		✓		✓
Neil	Scotland	Male	Caucasian	3	Yes	✓	✓	✓	✓	✓		
Craig	Scotland	Male	Caucasian	3	Yes				✓	✓	✓	✓
Ashton	Scotland	Male	Caucasian	2	No			✓	✓			
Simon	Scotland	Male	Caucasian	2	No				✓		✓	✓
Claire	Scotland	Female	Caucasian	3	Yes	✓	✓	✓	✓	✓		✓
Henry	Scotland	Male	Caucasian	1	No				✓	✓	✓	✓
Mary	Scotland	Female	Caucasian	2	No			✓		✓		
Conor	Scotland	Male	Caucasian	2	No	✓	✓	✓				

### IPA Findings

Three overarching group experiential themes, ‘*everything was just starting to fall into place’*, ‘*caught in a trap’*, and ‘*trials and tribulations*’, together with the seven subordinate group experiential themes, convey the structure and essence of participants’ experiences of personal recovery in relation to temporary accommodation. Collectively, those themes reveal a duality of influence of temporary accommodation on recovery. Those ambiguous spaces confronted participants with existential uncertainty and fear, volatility and chronic boredom, but also proffered opportunities for envisioning and enacting recovery.

#### ‘Everything Was Just Starting to Fall into Place’: Recovery-Enabling Role of Temporary Accommodation

##### ‘Peace of Mind’: Restoring ‘Homelikeness’

The overwhelming majority of the participants described their lives ‘before’ as a chronic state of ‘*unhomelikeness*’ (Svenaeus, 2000) pervaded by adverse life events such as bereavement, incarceration, institutionalisation, housing instability, unemployment and domestic violence, which, in many cases, had led to a state of short-termism, existential disorientation, lack of progress and inertia:

*‘Moving all the time, not knowing the future or anything. You've* [got] *no security wherever you are.* […] *I'd wake up in the morning and I saw myself just...going nowhere* […]*’* (Neil)

While several participants emphasised the enduring impact of chronic life adversity, many highlighted that entering their current temporary accommodation demarcated a turning point. Specifically, they had acquired stability and security, which facilitated their contemplative, self-management and goal-setting practices, all of which aided recovery. As Scott, a Caucasian safe haven client in his late 50s, shared:

*‘I started to feel more secure with where I was and what was going on around me.’* (Scott)

Neil, an assessment centre client in Scotland in his late 50s who had been homeless for six years, for instance, reflected:

‘Interviewer: *What do you think is the main factor that helped you feel better now?*

Neil: *This place. My life changed for the better straightaway*. [...] *You're looked after, you are secure.* [...]’

**Figure 1.**
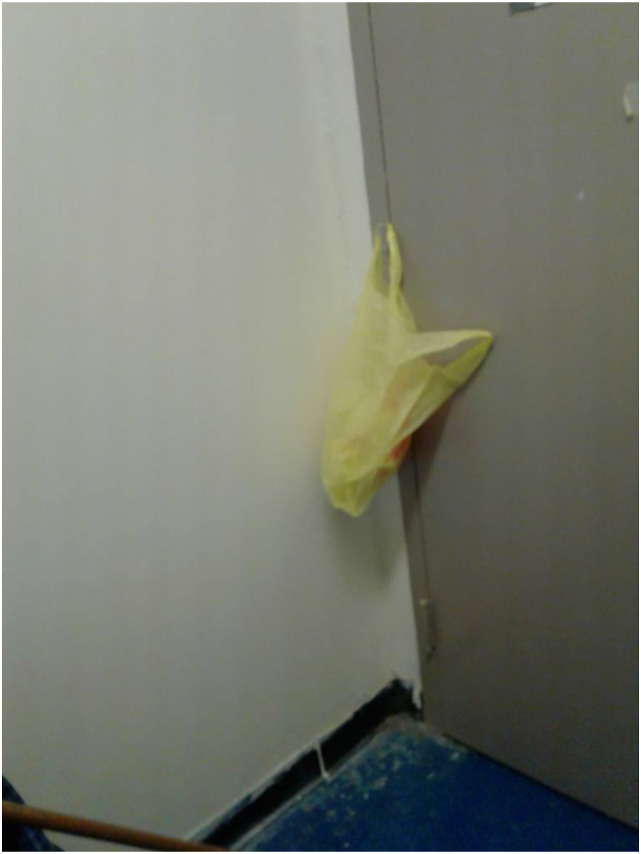
A plastic bag hanging on Benjamin’s room door.

Participants such as Benjamin, Neil and Liam shared that they felt a sense of relief, gratitude, peace and ‘*easing*’ of the mind. In an audio diary entry, Benjamin, an African-American safe haven client in his late 50s who had spent 30 years being homeless in his lifetime, highlighted having secure housing as a crucial enabler of his well-being and autonomy. Benjamin also took a photograph of a plastic bag hanging on his room door (See ‘Figure 1’). The depiction of the bag, a seemingly mundane object, could be interpreted as Benjamin’s gradual restoration of ‘*homelikeness*’. The photograph was taken in response to the diary question: ‘*What is something that best captures your life now?’*:

*‘Well, I’m here in this shelter right now. The shelter is called ‘a safe haven’. You’ve got your own room. And by me having my own room-that gives me peace of mind* […] *you’re safe.* [...] *I mean it's better than where I was at anyway. Because once upon a time, I didn't have no door to hang no bag on-you see what I'm saying? I didn't have no door to close. I'm saying it's the little things you gotta be grateful for.’*

Benjamin’s account shows his increased mindfulness, control over his environment and sense of ownership.

‘Homelikeness’ emerged not just from having a physical shelter and access to instrumental assistance, but also from providers’ gestures and everyday acts of caring. Edward, a Hispanic drop-in centre client in his late 50s who had been homeless for five years in his lifetime, for instance, shared how his case worker’s helpful and approachable demeanour had impacted positively on his mental well-being:

‘*The way she treats me, she talks to me - more like a family member.* [...] *I could just talk to her about anything. She will just break me out...I even forget that I was depressed.’*

##### A Place for Reflection and Insight

The re-establishment of the sense of security, constancy and ‘homelikeness’, in turn, appeared to enhance several participants’ capacities for reflection, self-knowledge and insight – catalysing recovery. Conor, a temporary accommodation resident in Glasgow in his late 20s who had been homeless for a total of three years, for example, described the transformational experience of a renewed sense of control and autonomy as a result of the safety, security and support he had received at his current provider:

‘*The last couple of months have been a hell of a wake-up call […] I thought that everything was just starting to fall into place*.’

Since moving into temporary accommodation, Conor had had access to regular support from accommodation staff, psychiatric and substance use services. As a result, he felt he had ‘*a clear head*’, was able to *‘think for himself*’ and re-evaluate his priorities, and was ‘*on the road to*’ normality.

Scott, who had had mental health difficulties from an early age and had spent five years without a home, described his life ‘*on the streets*’ as being preoccupied with maladaptive coping behaviours (for example, *‘looking for the quick fix’* and self-medicating) and the need for survival:

*‘When I was out on the streets, I acted before I thought. And that's what got me in a lot of trouble…* [...] *I didn't have to think about my real problems.* [...] *Because I was too occupied with all the negative behaviours, the survival skills…’*

In contrast, moving into temporary housing facilitated access to professional support and to a routine, which helped him ‘*take life a day at a time*’ and adopt ‘*positive*’ behaviours such as adhering to medication, trusting his providers and re-evaluating his past. Engaging with his social worker, with psychotherapy and with other services enhanced his self-awareness and self-acceptance. This lifestyle change brought about insight and a commitment to achieving a better life:

*‘I needed a life that I could achieve small goals. A life that was simple on a daily basis. I had to learn how to take life a day at a time.* [...] *I first had to understand why I was feeling the way I was feeling…* [...] *I knew that I didn't want a reckless life.’*

##### Occupational and Social Engagement

**Figure 2.**
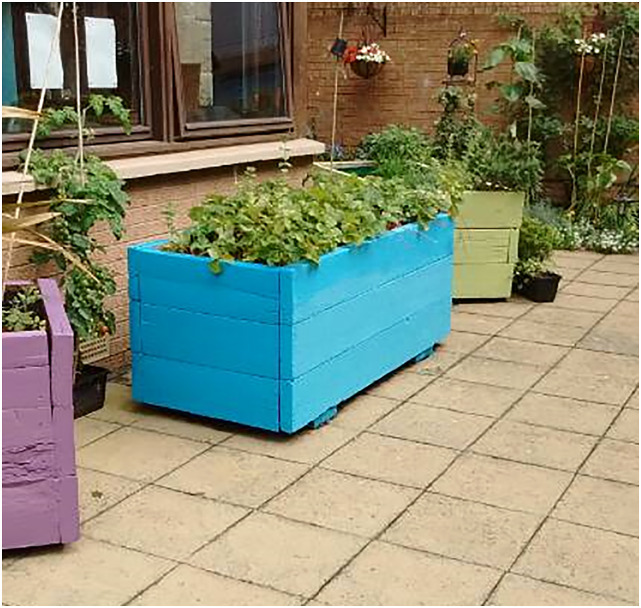
A photograph taken by Claire showing the gardening activities that she enjoyed.

Temporary accommodation also proffered opportunities for occupational, recreational and social engagement – facilitating recovery. For several participants such as Claire, a Scottish woman in her late 30s who had been homeless for five years in her lifetime, ‘*doing’* helped create structure and a sense of progress. For Claire, the chronic inactivity induced feelings of hopelessness, helplessness and depression (the *‘black hole’*), which were ameliorated by *‘being out and about’* – for instance, participating in arts and crafts or communal gardening activities (See ‘Figure 2’):

*‘I like doing stuff that takes your mind away from it. If you focus on something, it takes your depression mode away. […] It feels good because you've done/you've achieved something, you've done something so it's good […] it was a...therapy […]’*

Several other participants reflected on the therapeutic value of leisure activities organised by accommodation staff such as trips and arts-based activities.

#### ‘Caught in a Trap’: Recovery-Impeding Role of Temporary Accommodation

##### A Place of Illness

Several participants (for example, Claire; Mary; Craig; Kelly; Susan) discussed the volatility of their temporary accommodation as a significant barrier to coping with mental illness. Mary, a Scottish woman in her late 30s who had been homeless for a total of 17 years in her lifetime, for instance, shared that the transitory nature of the shelter engendered a sense of insecurity and lack of control, which exacerbated her mental health difficulties:

*‘[…] It's quite a volatile place — it's no (?) safe! […] Your mood, everything is always up and down-especially in these places.’*

Other participants such as Susan, Kelly and Craig recounted traumatising experiences in homeless shelters, and described how the social environment of the shelter had induced depression and anxiety.

For the three drop-in centre participants, Oliver, Susan and Edward, the day centre environment was often unpredictable and volatile, which was extremely taxing – both physically and emotionally:

*‘I'm just...hanging in there until I get my place. And it would be more easy for me. I would not have so much headaches...My mind won't be like it wants to explode... […].’* (Edward)

The metaphoric verbs *‘hanging’* and *‘explode’* convey this participant’s embodied lack of control over the environment.

##### ‘Open Prison’

In contrast to the NYC participants, many of whom occupied congregate living spaces, the narratives of several Scottish participants, all of whom resided in single-occupancy rooms, were pervaded by chronic boredom. Neil, for instance, experienced boredom as an overwhelming sense of entrapment. For him, boredom was a socially and psychologically depriving experience, which he compared to an ‘*open prison*’:

*‘It's not like being in prison because you can go out. But it's similar to being in an open prison.* […] *The four walls* — *that's what leads me into trouble.*’

This extreme metaphor reflects Neil’s restricted opportunities to engage in occupational and recreational activities due to his homelessness, social isolation, physical disability and financial difficulties. Neil’s mobile phone diary images – mostly taken in his room – depicted him as a passive bystander to life ‘outside’. When asked via the mobile phone diary what something that made his situation worse was, he replied:

‘*Depression, boredom, alcohol.*’

Similarly, Craig’s present-day narrative revealed a state of passivity and hopelessness, in which *time* was experienced as painful and entrapping:

*‘Boredom. Just trying to kill the day and night away. It's basically all you do every day, all day-just...kill time. Every morning that I wake up, I can't wait until the next time I have to go to sleep again...just...try to get away with the days […] I'm kinda caught in that trap.’*

**Figure 3.**
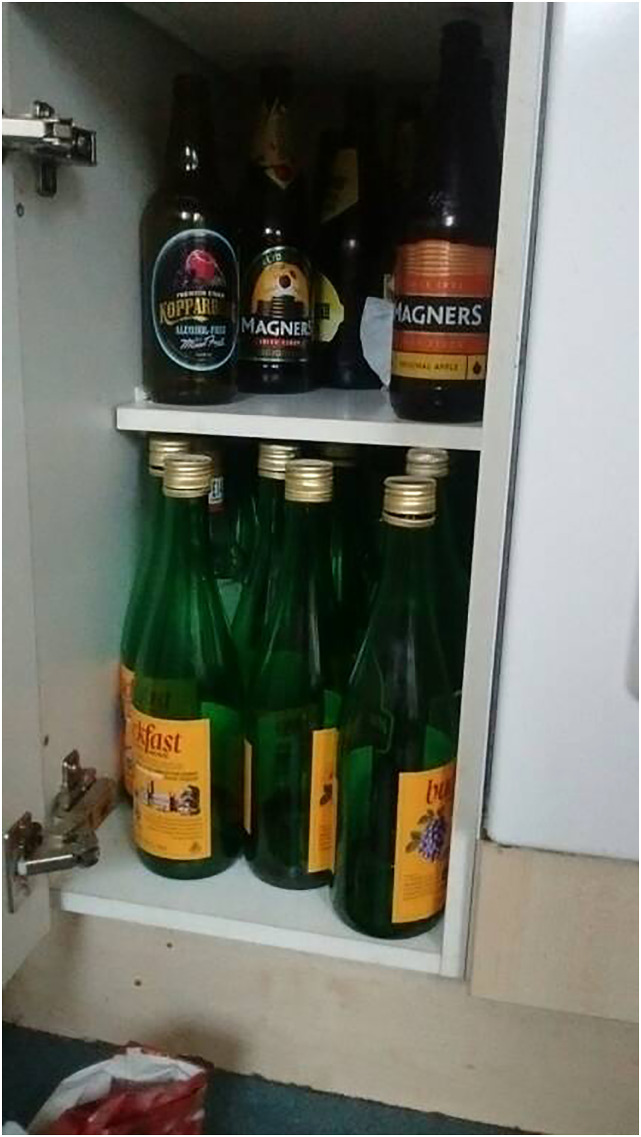
An image generated by Craig, with the caption ‘have nothing to do and nowhere to go’.

‘Figure 3’ depicts a photograph Craig, an assessment centre client in his late 40s who had been homeless for two years in his lifetime, took of empty liquor bottles – signifying the painful absences, disappointments and unfulfilled needs and wants that pervaded his existence.

**Figure 4.**
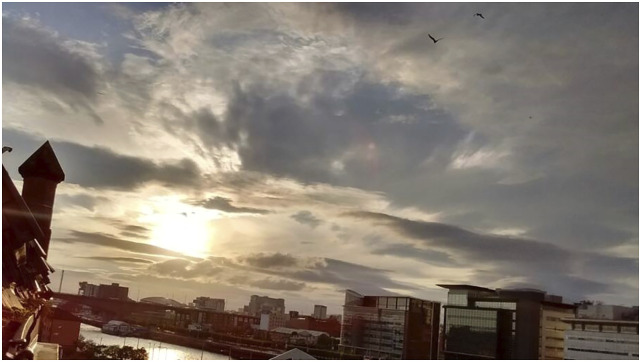
An image by Craig depicting the view from his room at the shelter.

In another photograph, Craig depicted the view from the shelter (‘Figure 4’). The numerous images Craig generated of him looking out from his room at the shelter seemed to symbolise his sense of forced passivity and social exclusion.

##### ‘Existential Vacuum’

**Figure 5.**
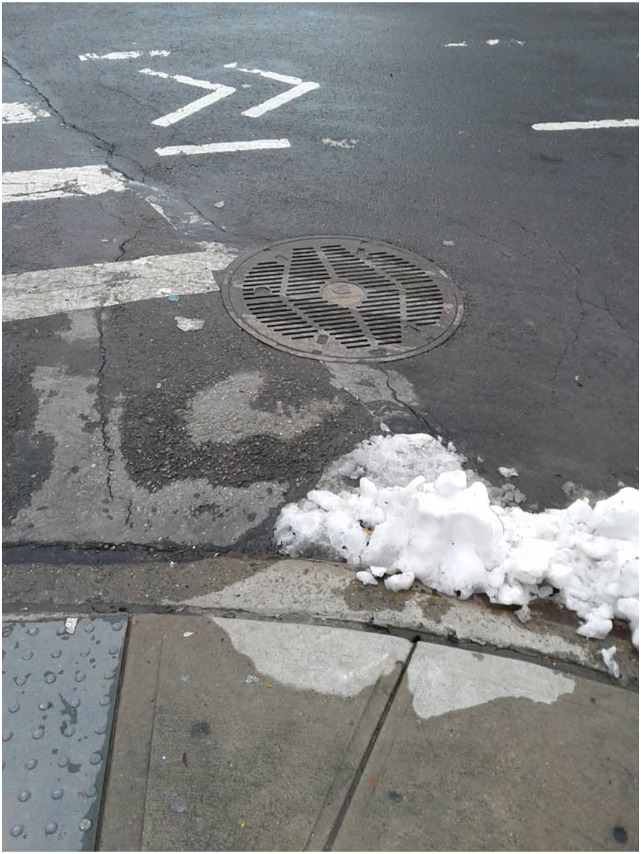
A photograph of a drain basin obtained by Matthew.

More profoundly, the narratives of shelter living and homelessness by participants such as Matthew, Craig and Henry featured emotive accounts of their daily battles with meaninglessness and the sense of the unavoidability of suffering – revealing a deepening state of ‘*existential vacuum’* (Frankl, 1969). Matthew, a safe haven client in his late 50s, felt indescribable and paralysing anguish, hopelessness and ‘*disappointment*’. Under the burden of chronic homelessness, food insecurity, multiple health concerns and unresponsive health and social care systems, Matthew felt marginalised and discarded from society – economically and symbolically – captured by a photograph of a drain basin (‘See Figure 5’):

*‘Feeling kinda like…discarded.* […] *I don’t know* — *I just…I feel depressed.* […] *I’ve nothing. It’s like a daily struggle.* [...] *Honestly, I'm tired of the struggle of trying to reach out and ask for help.’*

Matthew’s account revealed his painful sense of separateness from others – an experiential state of emptiness and ‘*non-being’* (McGraw, 1995).

Henry was an assessment centre client in his late 40s who had been homeless for five years in his lifetime. Henry’s account also revealed a sense of entrapment and anguish as he felt deprived of social connections, financial security and housing stability:

*‘It's just...I'm here and...what else have you got to do...? ((voice becomes weaker)) There's nothing else you can do apart from just wait and bide your time* [...]*’*

The rhetorical question and non-verbal cues betray Henry’s emotional suffering triggered by his lack of control over his living conditions and the uncertain future.

### ‘Trials and Tribulations’: Temporary Accommodation as an Ambivalent Site for Recovery

The final group experiential theme, ‘*trials and tribulations’*, captures the ambivalent role of temporary accommodation in participants’ mental well-being and recovery, in that living in the shelter was perceived as a source of both psycho-emotional and existential suffering *and* meaning and purpose.

Craig, for instance, described his current circumstances as a *‘juncture’* – a metaphor imbued with both uncertainty and disorientation *and* possibilities for an alternative life – a phenomenon which McGraw (1995) defines as a ‘*boundary situation*’ – conditions that are often emotionally and existentially challenging and difficult to comprehend or rationalise, but that are ‘[…] *potential turning points in one's life and are mandatory for the development of authenticity.*’ (p. 57):

‘*So I am kinda...50/50* […] *I am kinda stuck right here in this kinda...‘don't know’ what's gonna happen.’*

The dilemmas of meaning several participants faced on a daily basis – the fragility and contingency of hope amidst homelessness – were poignantly exemplified by Oliver, an African-American drop-in centre client in his late 50s who had been homeless for four years, who described the plight of a fellow day centre client:

*‘She is sitting there every day like that. […] She sits there like that, like she is just waiting to shut her eyes and that's it.* [...] *She is grieving. […] She just wants to give up. […] I told her, you know: ‘It's not over.’ You're here for a purpose […] That can happen to anybody just like that and I can be in her shoes […].’*

Oliver, who had felt hopeless before but managed to ‘*come out on top’,* emphasised the importance of faith and perseverance amidst the existential threats of homelessness and co-occurring life adversity.

Interestingly, two U.S. participants, Scott and Oliver, described their life hardship as their *‘trials and tribulations’:*

*‘I mean, it's very difficult but as I'm going through my trials and tribulations that I've been going through, other people out there cheer me so I can cheer somebody else up*.’ (Oliver)

This Biblical metaphor could be interpreted as alluding to those participants’ stoicism and faith. Both Scott and Oliver shared that achieving recovery, positive well-being and rehousing required daily labour, which involved withstanding the stresses and strains of homelessness. Oliver, for instance, shared his experience of losing *‘patience*’ as a result of what he perceived was unfairness and lack of transparency of the rehousing process. Scott interpreted his predicament as necessary for his ‘*growth*’ and ‘*maturity*’. Those participants appeared to ‘*emplot*’ their hardship as ‘*suffering towards’* a higher state of being (McGraw, 1995, citing Frankl, 1969).

Oliver found meaning in *‘hanging on’* and helping others:

‘[...] *Even if somebody is down, we try to lift them up, you know, by talking to them, make them laugh, say something funny. In this room, we try to keep the atmosphere OK. We don't want it to be really a sad, sad atmosphere. We try to bring it up. 'Listen, you still got love in this room no matter what happened.’*

Oliver’s account reveals his efforts to co-create an atmosphere of togetherness, hope and resilience. It seems the residents’ shared (human) vulnerabilities bound them together and gave more meaning to their adversity. Even amidst existential uncertainty, several participants sought and forged routes to wisdom and personal growth.

## Discussion

This transatlantic investigation contributes to the dearth of research, particularly in the Scottish context, on the relationship between mental well-being, recovery and temporary accommodation in chronic homelessness. The combination of repeat in-depth interviewing and a pluralistic data collection strategy featuring a novel mobile phone diary allowed this phenomenological study to capture a range of dynamic, situated experiences – demonstrating how changes in experiences of place, time and self impacted participants’ recovery capacities (Duff, 2012). Harnessing the expressive capabilities of the multi-modal diary, several participants articulated intimate feelings and experiences such as gratitude and hope but also isolation and existential fear. Oftentimes, the diaries yielded novel themes not discussed during the initial interviews. The photographs, in particular, evocatively depicted various contextual hinderers and facilitators of mental health recovery. For instance, they revealed how seemingly mundane objects, activities and encounters had symbolic significance for participants’ recovery journeys. The audio entries, on the other hand, conveyed participants’ in-situ commentaries with immediacy and rich contextual detail. Therefore, the diary and the elicitation interviews helped access participants’ *tacit knowledge* – the taken-for-granted, quotidian and implicit aspects of daily life (Tracy, 2010). As also illustrated in Piat et al.'s (2017) photo-elicitation study into formerly homeless adults in supported housing, the present study showed how the affective and symbolic properties of everyday practices and spaces deeply affected mental well-being and recovery. This adds empirical support for Piat et al.'s (2017) view of recovery as ‘*an ecological and situated process*’ (p. 77).

Furthermore, the repeat interviewing facilitated rapport-building and disclosure – a crucial challenge to researching sensitive topics and vulnerable groups (Dickson-Swift et al., 2007). It also enabled the researcher to interrogate and clarify any ambiguities and contradictions in the data, and corroborate central findings across interviews and across modalities. This enhanced the depth, nuance and credibility of the analysis (Smith et al., 2022; Tracy, 2010).

The present study demonstrates that, for clients with a history of chronic homelessness, temporary housing can, indeed, promote recovery. Several participants’ positive experiences of temporary housing – which were not altogether unexpected given the specialised support offered by the safe havens and the Scottish emergency housing providers – largely cohere with Lincoln et al.'s (2009) findings regarding the increased privacy and autonomy reported by safe haven clients, and contributes to the relatively small body of literature on the positive impact of temporary housing in clients with complex needs (Kerman & Sylvestre, 2020; Phipps et al., 2017).

The present findings also resonate with, and expand upon, Padgett et al.'s (2022) recent findings of the positive effects of hotel stays on previously unsheltered individuals’ mental and emotional well-being in the context of COVID-19, specifically the sense of safety, peace and a ‘*mental space for future planning*’ (p. 5). In contrast, the present study focuses exclusively on clients with complex needs and offers a more balanced analysis of both recovery-promoting and recovery-impeding aspects of temporary accommodation. It is possible that the more naturalistic and participant-centred mobile phone diary and visual elicitation in the current study helped minimise any social desirability bias and prompt reflections on previously taken-for-granted aspects of daily life – eliciting more nuanced accounts (O’Connell, 2013). Another possible reason for the more nuanced findings in the present study is its focus on participants’ *in-situ* experiences compared to research gathering former residents’ retrospective accounts (for example, Eaton et al., 2022).

Conversely, the present findings of the recovery-impeding functions of temporary accommodation build on prior studies on clients’ negative shelter experiences (Meanwell, 2012; Kerman & Sylvestre, 2020; Bonugli et al., 2013). Unlike Kerman and Sylvetre’s (2020) broader focus on ‘*health and social services*’, including mental health services, this study focused specifically on the experiences of temporary accommodation and its associated provisions. As such, it provides more contextualised and detailed insights into the relationship between recovery and concrete housing provisions and atmospheres.

The present study offers empirical support for the transformational, recovery-promoting potential of temporary accommodation in clients with complex needs proposed in other studies (Bonugli et al., 2013; Kerman & Sylvestre, 2020). Yet, the differential impact of temporary accommodation on participants’ recovery evident in this study indicates that the functions of the shelter and its facilities are not fixed and universal, but depend on the extent to which they afford ‘*discrete enabling resources’* (material, affective, relational) in relation to each client’s evolving perspectives, goals and values (Doroud et al., 2018; Duff, 2012: p. 1388).

### Limitations

First, several subgroups were underrepresented – including homeless youth, women, families, Caucasian Americans and Black and minority ethnic Scottish individuals. The underrepresentation of women is a significant limitation given the research on the distinct challenges experienced by homeless women, including mothers (Finfgeld-Connett, 2010). Second, gathering service providers’ views would have helped generate an even more nuanced, multi-perspectival analysis (See Phipps et al., 2017, for an example). Third, attrition resulted in uneven interview completion rates. This, together with the low uptake of the mobile phone diary, affected the completeness of the data. The observed attrition rates were not altogether unexpected given participants’ precarious living conditions, multiple, complex needs and busy schedules including housing applications, work-related occupations and appointment attendance. Lastly, longitudinal qualitative investigations offer greater promise for unravelling the situated and dynamic experiences of (non-)recovery along the housing continuum (Lincoln et al., 2009).

### Recommendations for Policy, Research and Practice

The recovery-impeding effects of the shelter illustrated in this study support the calls for minimising reliance on temporary accommodation and ensuring clients’ rapid access to permanent housing that is commensurate to their needs (Sanders & Reid, 2018; Wusinich et al., 2019). This requires further investments in client-centred housing programmes, such as Housing First, and in more affordable housing in both Scotland and the U.S. (Sanders & Reid, 2018; Wusinich et al., 2019). In the meantime, as prolonged residency at temporary accommodation remains a reality for many, embedding a recovery orientation in those service settings through advocacy, staff training, service leadership, cross-service collaboration and user involvement in service design and delivery will mitigate against erecting ‘*additional barriers, remarginalizing the most vulnerable*’ (Kerman & Sylvestre, 2020; O'Shaughnessy & Greenwood, 2021; Quirouette, 2016: p. 317).

A humanising ethos of care informed by each client’s unique social positionality, biography, strengths and aspirations is imperative in supporting clients’ long-term recovery and resettlement journeys (Phipps et al., 2017; Todres et al., 2009; Topor et al., 2021). For instance, providers should be cognizant of how the multiplicity of biographical and structural forms of disadvantage may give rise to existential uncertainty, apathy and hopelessness, which could be mislabelled as service resistance or non-compliance (Padgett et al., 2016; Phipps et al., 2017). Such a person-centred service culture should be combined with expanding clients’ access to vocational opportunities such as skills development programmes, volunteering, community projects and other employment support (Sanders & Reid, 2018; Marshall et al., 2017; Doroud et al., 2015); in addition to recreational activities, which may have a range of therapeutic, spiritual and socialisation benefits (Nicholson Goertzen & Litwiller, 2021). More research is warranted into how various occupations and service contexts help foster recovery-relevant processes such as self-expression, self-knowledge and community belonging and participation (Doroud et al., 2015).

Maximising opportunities for positive social interactions in the shelter, while preserving each client’s sense of safety, privacy, control and autonomy, is also recommended (Petrovich et al., 2017). For instance, Petrovich et al. (2017) document an array of low-cost modifications to the shelter space that could be conducive to day centre clients’ safety and privacy, dignity, a sense of community and well-being – from personalising interior settings through flexible seating arrangements and artwork displays, through to space features and practices that promote trust and respect between staff and clients. Importantly, such spaces should be co-designed with clients to reflect their evolving well-being needs and recovery journeys (Doroud et al., 2018).
